# Integrating audio and visual modalities for multimodal personality trait recognition *via* hybrid deep learning

**DOI:** 10.3389/fnins.2022.1107284

**Published:** 2023-01-06

**Authors:** Xiaoming Zhao, Yuehui Liao, Zhiwei Tang, Yicheng Xu, Xin Tao, Dandan Wang, Guoyu Wang, Hongsheng Lu

**Affiliations:** ^1^Taizhou Central Hospital (Taizhou University Hospital), Taizhou University, Taizhou, Zhejiang, China; ^2^School of Computer Science, Hangzhou Dianzi University, Hangzhou, China; ^3^School of Information Technology Engineering, Taizhou Vocational and Technical College, Taizhou, Zhejiang, China

**Keywords:** multimodal personality trait recognition, hybrid deep learning, convolutional neural networks, bi-directional long short-term memory network, Transformer, spatiotemporal

## Abstract

Recently, personality trait recognition, which aims to identify people’s first impression behavior data and analyze people’s psychological characteristics, has been an interesting and active topic in psychology, affective neuroscience and artificial intelligence. To effectively take advantage of spatio-temporal cues in audio-visual modalities, this paper proposes a new method of multimodal personality trait recognition integrating audio-visual modalities based on a hybrid deep learning framework, which is comprised of convolutional neural networks (CNN), bi-directional long short-term memory network (Bi-LSTM), and the Transformer network. In particular, a pre-trained deep audio CNN model is used to learn high-level segment-level audio features. A pre-trained deep face CNN model is leveraged to separately learn high-level frame-level global scene features and local face features from each frame in dynamic video sequences. Then, these extracted deep audio-visual features are fed into a Bi-LSTM and a Transformer network to individually capture long-term temporal dependency, thereby producing the final global audio and visual features for downstream tasks. Finally, a linear regression method is employed to conduct the single audio-based and visual-based personality trait recognition tasks, followed by a decision-level fusion strategy used for producing the final Big-Five personality scores and interview scores. Experimental results on the public ChaLearn First Impression-V2 personality dataset show the effectiveness of our method, outperforming other used methods.

## 1. Introduction

In personality psychology, researchers believe that human personality is innate, and have developed various theoretical methods to understand and measure a person’s personality. Costa and McCrae (1998) proposed a personality trait theory, in which personality characteristic were referred to as the main factors affecting the characteristics of individual behaviors, the critical factor in forming personality traits, and the basic unit for measuring personality traits. In Vinciarelli and Mohammadi (2014) personality is defined as: “personality is a psychological construct that can explain the diversity of human behaviors on the basis of a few, stable and measurable individual characteristics.” At present, researchers have used psychological scales to establish various personality traits models, including Big-Five (McCrae and John, 1992), Cattell sixteen personality factor (16PF) (Karson and O’Dell, 1976), Myers-Briggs type indicators (MBTI) (Furnham, 1996), Minnesota multiple personality inventory (MMPI) (Bathurst et al., 1997), and so on. Among them, the Big-Five model has become the most fashionable measure model for automatic personality trait recognition. In particular, the Big-Five model, also known as the OCEAN model, aims to measure a person’s personality through five dipolar scales: openness (O), conscientiousness (C), extroversion (E), agreeableness (A), and neuroticism (N). In affective neuroscience, the neural mechanisms of emotion expression are investigated by means of combining neuroscience with the psychological study of personality, emotion, and mood (Montag and Davis, 2018; Wang and Zhao, 2022; Zhang et al., 2022).

In recent years, researchers have employed computational techniques such as machine learning and deep learning methods (Gao et al., 2020; Liang et al., 2021; Wang and Deng, 2021; Yan et al., 2021; Ye et al., 2021) to model and measure human personality from the first impression behavior data, which is called personality computing (Junior et al., 2019). One of the most important research subject in personality computing is automatic personality trait recognition, which aims to identify people’s first impression behavior data by computer and then analyze people’s psychological characteristics (Zhao et al., 2022). Personality trait recognition has significant applications to human emotional behavior analysis, human-computer interaction, and interview recommendation. For example, Zhao et al. (2019) explored the influence of personality on emotional behavior by means of a hypergraph learning framework. When an enterprise recruits, human resource department can leverage personality trait recognition techniques to analyze personality characteristics of the job seekers by collecting their first-impression behavior data, and then select employees who can better meet the needs of the enterprise. To advance the development of personality trait recognition, the 2016 European Conference on Computer Vision (ECCV) released a publicly available personality dataset, i.e., ChaLearn-2016, and organized an academic competition of personality trait recognition (Ponce-López et al., 2016). Since 2016, personality trait recognition has become a hot research topic in psychology, affective neuroscience, and artificial intelligence.

In a basic personality trait recognition system, two important steps are involved: feature extraction and personality trait classification or prediction (Zhao et al., 2022). Feature extraction aims to derive appropriate feature parameters related to the expression of personality traits from the acquired first impression behavioral data. Personality trait classification or prediction aims to employ machine learning methods to conduct personality classification or prediction. The conventional classifiers or regressors such as support vector machines (SVM) and linear regressors can be adopted for personality trait classification or prediction. This paper will focus on feature extraction in a personality trait recognition system.

According to the types of extracted features characterizing personality traits, personality trait recognition techniques can be divided into hand-crafted based methods and deep learning based methods. Based on the extracted hand-crafted or deep learning features, previous works (Zhao et al., 2022) focus on performing personality trait recognition from single modality, such as audio-based personality trait recognition (Mohammadi and Vinciarelli, 2012), visual-based personality trait recognition (Gürpınar et al., 2016), etc. Although these works based on single modality have achieved good performance, there are still two limitations for them. First, the people’s first impression behavior data in real-world scenery are often multimodal rather than single-modal for characterizing personality traits. For instance, both verbal and non-verbal information such as audio and visual modality are highly correlated with personality traits. In this case, it is thus necessary to adopt multiple input modalities for personality trait recognition. Second, although deep learning methods have been fashionable for personality trait recognition, each of them has its advantages and disadvantages. Therefore, integrating the advantages of different deep learning methods may further improve the performance of personality trait recognition, which will be investigated in this work.

**FIGURE 1:**
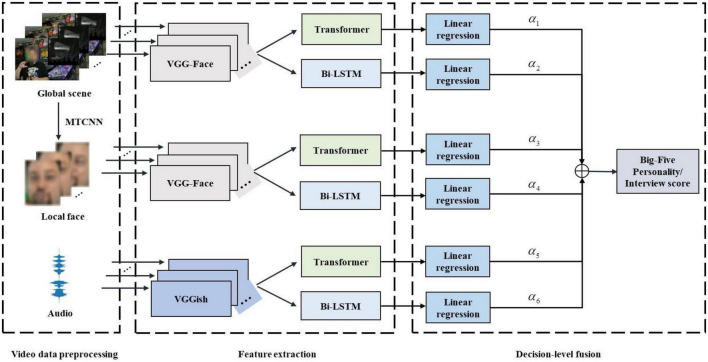
The flowchart of the proposed multimodal personality trait recognition method integrating audio and visual modalities based on a hybrid deep learning framework.

To address these two issues above-mentioned, this paper proposes a multimodal personality trait recognition method integrating audio and visual modalities based on a hybrid deep learning framework. As depicted in Figure 1, the proposed method combines three different deep models, including convolutional neural networks (CNN) (LeCun et al., 1998; Krizhevsky et al., 2012), bi-directional long short-term memory network (Bi-LSTM) (Schuster and Paliwal, 1997), recently emerged Transformer (Vaswani et al., 2017), to learn high-level audio-visual feature representations, followed by a decision-level fusion strategy for final personality trait recognition. In particular, for audio feature extraction, the pre-trained deep audio CNN model called VGGish (Hershey et al., 2017) is used to learn high-level segment-level audio features. For visual feature extraction, the pre-trained deep face CNN model called VGG-Face (Parkhi et al., 2015) is leveraged to separately learn high-level frame-level global scene image features and local facial image features from each frame in dynamic video sequences. Then, these extracted deep audio-visual features are fed into a Bi-LSTM and a Transformer network (Vaswani et al., 2017) to individually capture long-term temporal dependency, thereby producing the final global audio and visual features for downstream tasks. Finally, a linear regression method is employed to conduct the single audio-based and visual-based personality trait recognition tasks, and yield six independent personality trait prediction scores. A decision-level fusion strategy is adopted to merge these personality trait prediction scores and output the final Big-Five personality scores and interview scores. Extensive experiments is conducted on the public ChaLearn First Impressions-V2 dataset (Escalante et al., 2017), and demonstrate the effectiveness of the proposed method on personality trait recognition tasks.

The main contributions of this paper are summarized as follows:

(1) This paper proposes a multimodal personality trait recognition method integrating audio and visual modalities based on a hybrid deep learning framework, in which CNN, Bi-LSTM, and Transformer are combined to capture high-level audio-visual spatio-temporal feature representations for personality trait recognition.
(2) Extensive experiments are performed on the public ChaLearn First Impressions-V2 dataset and experimental results show that the proposed method outperforms other comparing methods on personality trait recognition tasks.

## 2. Related work

The majority of prior works for personality trait recognition concentrates on single modality such as audio or visual cues, as described below.

### 2.1. Audio-based personality trait recognition

In early works, the conventional extracted hand-crafted audio features are low-level descriptor (LLD) features including intensity, pitch, formants, Mel-Frequency Cepstrum Coefficients (MFCCs), and so on. Mohammadi and Vinciarelli (2012) derived the LLD features like intensity, pitch, and formants, and then employed a logistic regression to predict the Big-five personality traits in audio clips. An et al. (2016) extracted the typical Interspeech-2013 ComParE feature set (Schuller et al., 2013) and fed them into a SVM classifier to conduct the Big-Five personality trait recognition.

In recent years, researchers have tried to leverage deep learning (LeCun et al., 2015) models with a multilayer network structure to learn high-level audio feature representations for promoting the performance of personality trait recognition. Among them, the representative deep learning methods are CNN (LeCun et al., 1998; Krizhevsky et al., 2012), recurrent neural networks (RNN) (Elman, 1990) and its variants called long short-term memory (LSTM) (Hochreiter and Schmidhuber, 1997), etc. Hayat et al. (2019) proposed an audio personality feature extraction method based on CNN. They fine-tuned the pre-trained CNN model called AudioSet in the first-impression behavior dataset and extracted high-level audio features for Big-Five personality prediction, demonstrating the advantages of CNN-based learned features compared with hand-crafted features. Zhu et al. (2018) presented a method of automatic perception of speakers’ personality from speech in Mandarin. They developed a new skip-frame LSTM system to learn personality information from frame-level descriptor like MFCCs instead of hand-crafted prosodic features.

### 2.2. Visual-based personality trait recognition

In terms of the input type of visual data, visual-based personality trait recognition can be divided into two groups: static images-based and dynamic video sequences-based personality trait recognition.

For static images-based personality trait recognition, the extracted visual features mainly come from facial features, since facial morphology provides explicit cues for personality trait recognition. In early works, the commonly used hand-crafted facial features are color histograms, local binary patterns (LBP), global descriptor, aesthetic features, etc. Guntuku et al. (2015) extracted low-level hand-crafted features of facial images, including color histograms, LBP, global descriptor, and aesthetic features, and then employed the lasso regressor to predict the Big-five personality traits of users in self-portrait images. Recently, deep learning methods have been applied for static images-based personality trait recognition. Xu et al. (2021) explored the relationship between self-reported personality characteristics and static facial images. They investigated the performance of several deep learning models pre-trained on the ImageNet data, such as MobileNetv2, ResNeSt50, and the designed personality prediction neural network based on soft thresholding (S-NNPP) by means of fine-tuning them on the self-constructed dataset composed of facial images and personality characteristics.

For dynamic video sequences-based personality trait recognition, dynamic video sequences contain temporal information related to facial activity statistics, thereby providing useful and complementary cues for personality trait recognition (Junior et al., 2019). In early works, the hand-crafted video features related to facial activity statistics were usually adopted for personality trait recognition. Teijeiro-Mosquera et al. (2014) exploited the relationships between facial expressions in dynamic video sequences and personality impressions of the Big-Five traits. To characterize facial activity statistics, they extracted four kinds of behavioral cues for personality trait recognition, including statistic-based cues, Threshold (THR) cues, Hidden Markov Models (HMM) cues, and Winner Takes All (WTA) cues. Likewise, several recently developed deep learning methods have been employed for dynamic video sequences-based personality trait recognition. Gürpınar et al. (2016) extracted deep facial and scene feature representations in dynamic video sequences by fine-tuning a pre-trained VGG-19 model, and then input them into a kernel extreme learning machine to perform the prediction of Big-Five personality traits. Beyan et al. (2021) presented a classification method of perceived personality traits on the basis of novel deep visual activity (VA)-based features derived only from key-dynamic images in dynamic video sequences. They adopted a dynamic image construction, which aimed to learn long-term VA with CNN + LSTM, and detect spatiotemporal saliency to decide key-dynamic images.

## 3. The proposed method

To alleviate the problem of single modality based personality trait recognition, this paper proposes a multimodal personality trait recognition method integrating audio and visual modalities based on a hybrid deep learning framework. Figure 1 depicts the flowchart of the proposed method. As depicted in Figure 1, the proposed method adopts two modalities as its input: one is the audio signals, the other is the visual signals including the global scene images and facial images. The used hybrid deep learning framework comprises of three different deep learning models like CNN, Bi-LSTM, and Transformer, which are used for high-level feature learning tasks. The proposed method consists of three key steps: video data preprocessing, audio-visual feature extraction, and decision-level fusion, as described below.

### 3.1. Video data preprocessing

For audio signals in the video data, we use the pre-trained VGGish model (Hershey et al., 2017) to extract high-level audio segment-level features. It is noted that the length of speech segments as input of VGGish is required to be 0.96 s. To this end, the original audio signals in the video data are divided into to a certain number of adjacent segments which last a time period of 0.96 s.

For visual signals in the video data, two preprocessing tasks are implemented. For global scene images in a video, 100 scene images are selected at equal intervals form each original video sample. Then, the resolution of each global scene image is resampled from the original 1280×720 pixels to 224×224 as inputs of VGG-Face model (Parkhi et al., 2015). For local face images in a video, we employ the popular Multi-Task Convolutional Neural Network (MTCNN) (Zhang et al., 2016) to conduct face detection tasks. The resolution of face image detected in each frame is sampled to 224×224. Since some videos are affected by environmental factors such as illumination, MTCNN may detect face images with a low accuracy. As a tradeoff, 30 frames of detected face images are selected at equal intervals from the original video. For the video with less than 30 frames of detected face images, the first and last face images are repeatedly until the frame number of face video is 30.

### 3.2. Audio-visual feature extraction

Audio-visual feature extraction aims to learn the local and global feature representations from original audio and visual signals in a video for personality trait recognition, as described below.

#### 3.2.1. Audio-visual local feature extraction

For the divided audio segment with 0.96 s, we leverage the VGGish model (Hershey et al., 2017) pre-trained on the AudioSet dataset (Gemmeke et al., 2017) to capture high-level segment-level deep audio features. The used VGGish model consists of 6 convolutional layers, 4 pooling layers, and 3 fully connected layers. The kernel size of convolutional layers and pooling layers is 3×3 and 2×2, respectively. Since the neuron number of the last fully connected layer in the VGGish network is 128, the learned audio features by the VGGish model are 128-dimension.

For each scene and face image in a video, we employ the VGG-Face model (Parkhi et al., 2015) pre-trained on the ImageNet dataset (Deng et al., 2009) to learn high-level frame-level deep visual feature representations for downstream scene and face global feature learning tasks, respectively. The VGG-Face model includes 13 convolution layers, 5 pooling layers, and 2 fully connected layers. Since the neuron number of the last full connection layer in the VGG-Face network is 4096, the dimension of visual frame-level features obtained by VGG-Face network is 4096.

Given *i*-th input video clip *a*_*i*_ (*i* = 1,2,⋯*N*) and its corresponding Big-Five personality score *y*_*i*_, we fine-tune the pre-trained VGGish network (Hershey et al., 2017) to obtain deep segment-level audio feature representations, as described below:


(1) $$\min_{W^{V\,G},\theta^{V\,G}}\sum_{i=1}^{N}L\,\left(\text{sigmoid}\,\left(W^{V\,G}\,\eta^{V\,G}\,\left(a_{i};\theta^{V\,G}\right)\right),y_{i}\right)$$

where η*^VG^*(*a*_*i*_;θ*^VG^*) represents the output of the last full connected layer in the VGGish network. θ*^VG^* and *W^VG^* separately denotes the network parameters of the VGGish network and the weights of the sigmoid layer. The cross-entropy loss function *L* is defined as:


(2) $$L\,\left(V\,G,y\right)=-\sum_{j=1}^{N}y_{j}\,\log\left(y_{j}^{p}\right)$$

where *y*_*j*_ is the *j*-th ground-truth Big-Five personality score, and $y_{j}^{p}$ is represented by the predicted Big-Five personality score.

For deep visual scene and face feature extraction on each frame of video, we fine-tune the pre-trained VGG-Face network (Parkhi et al., 2015) to learn high-level visual feature representations. The process of fine-tuning the pre-trained VGG-Face network is similar to the above-mentioned Eqs 1, 2.

#### 3.2.2. Audio-visual global feature extraction

After completing the local audio and visual feature extraction tasks, it is necessary to individually learn the global audio features, visual scene features, and visual face features from the entire videos so as to conduct personality trait prediction tasks. To this end, we adopt the Bi-LSTM (Schuster and Paliwal, 1997) and recently emerged Transformer (Vaswani et al., 2017) to independently model long-term dependencies of temporal dynamics in video sequences, as described below.

Given an input sequence *e*_*t*_, the learning process of the Bi-LSTM network is:


(3) $$E=\mathrm{Bi}-\mathrm{LSTM}\,\left(W_{B\,i-L\,S\,T\,M},e_{t}\right)$$

where *E* ∈ ℝ^1×*d*^ is the learned temporal features, and *W*_*Bi–LSTM*_ is weight parameters of Bi-LSTM.

The original Transformer (Vaswani et al., 2017) is developed based on self-attention mechanisms like a Multi-Head attention without any recurrent structures and convolutions. A Multi-Head attention module consists of several Scaled Dot-Product Attention (SDPA) modules in parallel and then their outputs are concatenated as an input of a linear layer. Given the input query (*Q*), key (*K*), and value (*V*), the output of each SDPA module is defined as:


(4) $$\text{Attention}\,\left(Q,K,V\right)=\text{soft}\,\max\left(\frac{Q\,K^{T}}{\sqrt{d_{k}}}\right)\,V$$

where *d*_*k*_ is the feature dimension of the key matrix *K*.

### 3.3. Decision-level fusion

After obtaining audio-visual global features extracted by a Bi-LSTM model and a Transformer model, we adopt a linear regression layer to predict the Big-Five personality and interview scores. The linear regression layer is calculated as follows:


(5) $$f_{i}\,\left(x\right)=x_{i}\,w_{i}+b$$

where *x*_*i*_, *w*_*i*_, and *b* represent the *i*-th input sample, the corresponding weight value, and bias, respectively. *f*_*i*_(*x*) is the *i*-th prediction score value.

As shown in Figure 1, when using the learned audio features, visual scene features, and visual face features as inputs of a linear regression layer, we can obtain six different recognition results. To effectively fuse these six different recognition results, a weighted decision-level fusion strategy is employed, as described below:


(6) $$\tilde{f}\left(x\right)=\sum_{i=1}^{6}\alpha_{i}\,f_{i}\,\left(x\right)$$

where α_*i*_ is the weight value, *f*_*i*_(*x*) is the predicted value of each type of features, and $\sum_{i=1}^{6}\alpha_{i}=1$. The mean squared error (MSE) loss is computed as follows:


(7) $$\text{MSE}\,\left(\tilde{f}\left(X\right)\right)=E\,\left[{\left(\tilde{f}\left(X\right)-Y\right)}^{2}\right]=E\,\left[{\left(\sum_{i=1}^{6}\alpha_{i}\,\left(f_{i}\,\left(X\right)-Y\right)\right)}^{2}\right]$$

where *Y* is the ground-truth score. Our goal is to minimize the MSE loss subject to $\sum_{i=1}^{6}\alpha_{i}=1$. To this end, the Lagrangian expression of this problem is expressed as:


(8) $$L\,\left(X,\lambda \right)=\text{MSE}\,\left(\tilde{f}\left(X\right)\right)-\lambda \,\left(\sum_{i=1}^{6}\alpha_{i}-1\right)$$

where λ is the Lagrange multiplier.

Then, we calculate the partial derivation of Eq. 8 based on α_*m*_ for *m* = 1,2,⋯6, as defined as:


(9) $$\frac{\partial L\,\left(X,\lambda \right)}{\partial \alpha_{m}}=E\,\left[2\,\sum_{i=1}^{6}\alpha_{i}\,\left(f_{i}\,\left(X\right)-Y\right)\,\left(f_{m}\,\left(X\right)-Y\right)\right]-\lambda$$

We set the gradient to be 0, and get:


(10) $$2\,\sum_{i=1}^{6}\alpha_{i}\,E\,\left[\left(f_{i}\,\left(X\right)-Y\right)\,\left(f_{m}\,\left(X\right)-Y\right)\right]-\lambda =0,m=1,2,\cdots \,6$$

Let α = ^[α_1_,α_2_,α_3_,α_4_,α_5_,α_6_]*T*^, Ω = [*w*_*ij*_] = *E*[(*f*_*i*_(*X*)−*Y*)(*f*_*j*_(*X*)−*Y*)], Eq. 10 can be transformed as:


(11) $$\Omega \,\alpha =\frac{\lambda }{2}\,1$$

Then, the optimal weight vector α can be obtained by:


(12) $$\alpha =\frac{\Omega^{-1}\,1}{1^{T}\,\Omega^{-1}\,1}$$

## 4. Experiments

### 4.1. Dataset

**FIGURE 2:**
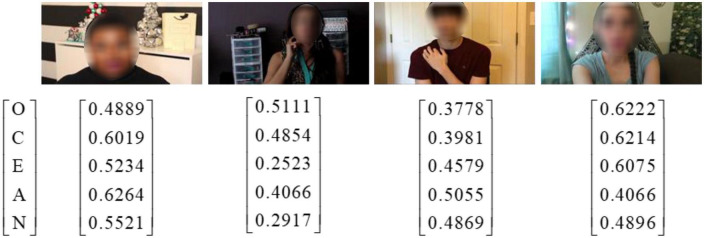
Image samples with the labeled Big-Five personality score from the ChaLearn First Impression-V2 dataset.

To verify the effectiveness of the proposed method, the public ChaLearn First Impression-V2 (Escalante et al., 2017) is employed for personality and interview prediction. This dataset contains 10,000 video clips collected from more than 3,000 different YouTube videos. The language involved in video participants is English. The resolution of the video is 1280×720, and the duration of each video clip is about 15 s. This dataset annotates the “Interview” scene labels for interview analysis. The divided training set, testing set and validation set in this dataset contain 6,000, 2,000, 2,000 video clips, respectively. In this work, we use the training and validation sets for experiments because the testing set is only open to competitors. Each video in this dataset is labeled by using the Big-Five personality score [0,1]. Figure 2 shows several image samples from the ChaLearn First Impression-V2 dataset.

### 4.2. Implementation details

When training all used deep learning models, the batch size is set to 32, and the initial learning rate is 1×*e*^−4^. After each epoch, the learning rate will become a half of the original learning rate. The maximum epoch number of is 30, and the Adam optimizer is used. The MSE loss function is adopted. The experimental platform is NVIDIA GPU Quadro M6000 with 24 GB memory. In order to improve the generalization performance of trained deep learning models and avoid overfitting, the early stopping strategy (Prechelt, 1998) is used.

In this work, we choose a two-layer Bi-LSTM to capture temporal dynamics related to video sequences. The number of neurons in each layer of Bi-LSTM is 2048. The number of encoding layer in the Transformer model is 6 for its best performance, and its last layer output 1024-dimension features. To compare with these deep learning models, several classical regression models such as Support Vector Regression (SVR) with polynomial (poly), radial basis function (RBF), and linear kernel functions, Decision Tree Regression (DTR) are employed. In the SVR model, the degree of polynomial kernel function is 3, the penalty factor “*C*” of radial basis kernel function is 2, and the parameter “gamma” is 0.5. The DTR model is implemented for its default parameters, such as the splitting policy “split = best” at each node, “min _ samples _ split = 2” for splitting an internal node. For these classical regression models, a simple average-pooling strategy is conducted on these extracted audio-visual local features so as to produce the global features as their inputs.

The evaluation metric for evaluating the predicted personality trait or interview scores is defined as:


(13) $$S=1-\sum_{j=1}^{N}\frac{\left|y_{j}^{p}-y_{j}\right|}{N}$$

where *N* is the number of samples, $y_{j}^{p}$ is the predicted value, and *y*_*j*_ is the ground-truth value. The higher the value *S* is, the better the obtained performance on personality or interview prediction tasks is.

### 4.3. Experimental results and analysis

In this section, two groups of experiments are carried out on the ChaLearn First Impression-V2 data set to verify the effectiveness of all used methods. One is the single-modal personality trait recognition, the other is multi-modal personality trait recognition.

#### 4.3.1. Results of single-modal personality trait recognition

For single-modal personality recognition, we present the experiment results and analysis based on the single extracted audio features, visual scene features, and visual face features by using the pre-trained deep models.

Table 1 shows the prediction results of deep audio features extracted by the pre-trained VGGish for different methods. “Transformer + Bi-LSTM” denotes that the learned features with Transformer and Bi-LSTM are directly concatenated to form a whole feature vector as inputs of the latter linear regression layer for prediction. It can be seen from Table 1 that Transformer + Bi-LSTM performs best based on deep audio features. More specially, the average Big-Five personality prediction score is 0.8952 and the corresponding interview prediction score of 0.8953, thereby outperforming other used methods. The ranking order for other used methods is Bi-LSTM, SVR (linear), SVR (rbf), Transformer, SVR (poly), and DTR. This shows the advantages of Transformer + Bi-LSTM on audio personality trait recognition tasks. It is noted that Transformer + Bi-LSTM performs better than Transformer and Bi-LSTM, indicating that there is a certain complementarity between Transformer and Bi-LSTM.

**TABLE 1:** Prediction results of deep audio features extracted by the pre-trained VGGish for different methods.

Models	O	C	E	A	N	Average score	Interview score
SVR (poly)	0.8540	0.8329	0.8624	0.8402	0.8744	0.8528	0.8319
SVR (rbf)	0.8967	0.8844	0.8932	0.9012	0.8906	0.8932	0.8920
SVR (linear)	0.8980	0.8846	0.8935	0.9025	0.8920	0.8941	0.8945
DTR	0.8541	0.8411	0.8542	0.8610	0.8453	0.8511	0.8511
Transformer	0.8972	0.8814	0.8920	0.9035	0.8907	0.8930	0.8915
Bi-LSTM	0.8986	0.8834	0.8932	0.9045	0.8928	0.8945	0.8947
Transformer + Bi-LSTM	**0.8989**	**0.8847**	**0.8938**	**0.9048**	**0.8935**	**0.8952**	**0.8953**

Bold values denote the highest performance.

Tables 2, 3 separately present personality prediction results of deep visual scene features and deep visual face features extracted by the pre-trained VGG-Face for different methods. It can be observed from Tables 2, 3 that Transformer + Bi-LSTM still obtains better performance other methods. In particular, Transformer + Bi-LSTM employs deep visual scene features and face features to produce the average Big-Five personality prediction scores of 0.9039 and 0.9124, respectively, and the interview prediction scores of 0.9057 and 0.9163, respectively. The ranking order for other used methods is Bi-LSTM, Transformer, SVR (poly), SVR (linear), SVR (rbf), and DTR. This shows the superiority of Transformer + Bi-LSTM on deep visual (scene and face) personality trait recognition tasks. The visual face images outperforms the visual scene images on personality trait recognition tasks. This may be because face images are more correlated with personality traits than scene images.

**TABLE 2:** Prediction results of deep visual scene features extracted by the pre-trained VGG-Face for different methods.

Models	O	C	E	A	N	Average score	Interview score
SVR (poly)	0.8921	0.8896	0.8896	0.8962	0.8850	0.8905	0.8890
SVR (rbf)	0.8841	0.8736	0.8804	0.8963	0.8780	0.8825	0.8818
SVR (linear)	0.8896	0.8872	0.8867	0.8922	0.8809	0.8873	0.8865
DTR	0.8636	0.8607	0.8627	0.8711	0.8586	0.8633	0.8639
Transformer	0.8941	0.8844	0.8909	0.9021	0.8884	0.8920	0.8920
Bi-LSTM	0.9042	0.9013	0.9012	0.9091	0.8993	0.9030	0.9050
Transformer + Bi-LSTM	**0.9043**	**0.9025**	**0.9035**	**0.9093**	**0.9000**	**0.9039**	**0.9057**

Bold values denote the highest performance.

**TABLE 3:** Prediction results of deep visual face features extracted by the pre-trained VGG-Face for different methods.

Models	O	C	E	A	N	Average score	Interview score
SVR (poly)	0.8871	0.8922	0.8923	0.8980	0.8855	0.8910	0.8963
SVR (rbf)	0.8841	0.8736	0.8804	0.8963	0.8780	0.8825	0.8818
SVR (linear)	0.8953	0.8922	0.8986	0.8974	0.8913	0.8950	0.8960
DTR	0.8714	0.8683	0.8702	0.8760	0.8674	0.8706	0.8721
Transformer	0.9023	0.9000	0.9029	0.9068	0.8968	0.9017	0.9017
Bi-LSTM	0.9103	**0.9155**	0.9129	0.9135	0.9085	0.9121	0.9161
Transformer + Bi-LSTM	**0.9110**	0.9148	**0.9130**	**0.9143**	**0.9087**	**0.9124**	**0.9163**

Bold values denote the highest performance.

In summary, the results in Tables 1–3 demonstrate that for single-modal personality recognition the visual face features perform best on personality trait and interview prediction tasks, followed by deep visual scene features and deep audio features. This shows that the facial images related to facial expression contain more discriminant information for personality trait recognition.

#### 4.3.2. Results of multimodal personality trait recognition

For multimodal personality recognition tasks, we compare the performance of three typical multimodal information fusion methods, such as feature-level fusion, decision-level fusion, and model-level fusion. In feature-level fusion, the audio-visual global features learned by Bi-LSTM and Transformer networks, are concatenated into a whole feature vector as input of the linear regression layer for personality trait prediction. In this case, feature-level fusion is also called early fusion (EF). In model-level fusion (MF), the concatenated audio-visual global features are fed into a 4-layer full-collection layer network (1024-512-256-128) for personality trait prediction. In decision-level fusion, we adopt Eq. 12 to obtain the analytical solution of the optimal weight values in Eq. 6. In this case, decision-level fusion is also called late fusion (LF).

Table 4 presents the comparisons of recognition results obtained by different fusion methods such as EF, MF, and LF, as well as the single modality methods. From the results in Table 4, we can see that: (1) among three used fusion methods, the used LF method combining audio, scene, and face obtains the best performance with an average score of 0.9167 on personality trait recognition tasks, and an average score of 0.9200 on interview prediction tasks. For personality trait recognition, the used EF method slightly outperforms the MF method, yielding an average score of 0.9154. By contrast, the used MF method slightly outperforms the EF method on interview prediction tasks. In particular, the MF method gives an average interview score of 0.9180. (2) All used fusion methods such as LF, MF, and EF provide superior performance to the single modality methods. This indicates the complementarity to some extent among audio, scene, and face modality on target recognition tasks.

**TABLE 4:** Comparisons of recognition results obtained by different methods.

Modality	O	C	E	A	N	Average score	Interview score
A	0.8989	0.8847	0.8938	0.9048	0.8935	0.8952	0.8953
S	0.9043	0.9025	0.9035	0.9093	0.9000	0.9039	0.9057
F	0.9110	0.9148	0.9130	0.9143	0.9087	0.9124	0.9153
A + S + F (EF)	0.9145	**0.9176**	0.9171	0.9158	0.9121	0.9154	0.9178
A + S + F (MF)	0.9151	0.9172	0.9156	0.9150	0.9123	0.9150	0.9180
A + S + F (LF)	**0.9167**	0.9163	**0.9176**	**0.9177**	**0.9150**	**0.9167**	**0.9200**

A, audio; S, scene; F, face; EF, early fusion; MF, model-level fusion; LF, late fusion. Bold values denote the highest performance.

#### 4.3.3. Comparisons with other existing methods

To further verify the effectiveness of the proposed method, Table 5 presents the comparisons of different used methods. Table 5 shows that the proposed method obtains an average score of 0.9167, which is better than other reported results obtained by audio, visual, and text modalities. This demonstrates the advantage of our method on personality trait recognition tasks. These comparing works are described as follows.

**TABLE 5:** Comparisons with other existing methods.

References	Modality	Feature extraction	Fusion methods	Average score
Güçlütürk et al., 2016	Audio, visual	Audio:ResNet-17 Visual:ResNet-17	EF	0.9109
Güçlütürk et al., 2017	Audio, visual, text	Audio:ResNet-17 Visual:ResNet-17 Text:skip-thought vectors	EF	0.9118
Wei et al., 2017	Audio, visual	Audio:MFCCs Visual:DAN	LF	0.9130
Principi et al., 2021	Audio, visual	Audio:1D CNN Visual:ResNet-50	MF	0.9160
Escalante et al., 2022	Audio, visual, text	Audio:ResNet-18 Visual:ResNet-18 Text: skip-thought vectors	LF	0.9161
Ours	Audio, visual	Audio:VGGish Visual:VGG-Face	LF	**0.9167**

EF, early fusion; MF, model-level fusion; LF, late fusion. Bold values denote the highest performance.

Güçlütürk et al. (2016) provided an audio-visual personality trait recognition based on 17-layer deep residual networks (ResNet-17). They concatenated the learned features of audio-visual streams at feature-level as an input of a fully connected layer and reported an average score of 0.9109 for final personality trait prediction. In this case, the used network does not need any feature engineering or visual analysis like face detection, face landmark alignment. Similarly, they also presented an multimodal personality trait analysis integrating audio, visual, and text modalities by using the 17-layer deep residual networks (Güçlütürk et al., 2017). Here, they extracted skip-thought vectors as text features. They fused these modalities at feature-level and reported an average score of 0.9118. Wei et al. (2017) presented a deep bimodal regression method of personality traits on short video sequences. For audio modality, they extracted MFCCs and logfbank features. For visual modality, they employed a modified CNN model called Descriptor Aggregation Network (DAN) to extract visual features. Finally, they fused these predicted regression scores of audio-visual modalities at decision-level, and reported an average score of 0.9130. Principi et al. (2021) presented a multimodal deep learning method integrating the raw audio and visual modalities for personality trait prediction. For audio modality, a 14-layer 1D CNN was used for audio feature extraction. For visual modality, they employed a pre-trained ResNet-50 network for visual feature extraction. Finally, they employed a fully connected layer to jointly learn audio-visual feature representations at model-level for final personality trait recognition, and achieved an average score of 0.9160. Escalante et al. (2022) proposed a multimodal deep personality trait recognition method based on audio, visual, and text modalities. They adopted a ResNet-18 to extract audio and visual features, and skip-thought vectors as text features. Then, a late fusion strategy was utilized to fuse all three modalities, and yielded an average score of 0.9161.

## 5. Conclusion

This paper presents a multimodal personality trait recognition method based on CNN + Bi-LSTM + Transformer network. In this work, CNN, Bi-LSTM, and Transformer are combined to capture high-level audio-visual spatio-temporal feature representations for personality trait recognition. Finally, we compare multimodal personality prediction results based on three different fusion methods such as feature-level fusion, model-level fusion, and decision-level fusion. Experiments on the public ChaLearn First Impression-V2 dataset show that decision-level fusion achieves the best multimodal personality trait recognition results with an average score of 0.9167, outperforming other existing methods.

It is noted that this work only focuses on integrating audio and visual modalities for multimodal personality trait recognition. Considering the diversity of modal information related to the expression of personality traits, it is interesting to combine current audio-visual modalities with other modalities such as physiological signals, text cues, etc., to further improve the performance of personality trait recognition. In addition, exploring a more advanced deep learning model for personality trait recognition is also an important direction in our future work.

## Data availability statement

Publicly available datasets were analyzed in this study. This data can be found here: https://chalearnlap.cvc.uab.cat/dataset/24/description/.

## Author contributions

XZ contributed to the writing and drafted the article. YL, ZT, YX, XT, DW, and GW contributed to the data preprocessing and analysis, software and experiment simulation. HL contributed to the project administration and writing—reviewing and editing. All authors contributed to the article and approved the submitted version.
